# Clonal relationships in recurrent B-cell lymphomas

**DOI:** 10.18632/oncotarget.7132

**Published:** 2016-02-02

**Authors:** Seung Eun Lee, So Young Kang, Hae Yong Yoo, Seok Jin Kim, Won Seog Kim, Young Hyeh Ko

**Affiliations:** ^1^ Department of Pathology, Konkuk University School of Medicine, Konkuk University Medical Center, Seoul, Korea; ^2^ Department of Pathology and Translational Genomics, Samsung Medical Center, Sungkyunkwan University School of Medicine, Seoul, Korea; ^3^ Department of Health Science and Technology, Samsung Advanced Institute for Health Sciences and Technology, Sungkyunkwan University School of Medicine, Seoul, Korea; ^4^ Division of Hematology and Oncology, Department of Medicine, Samsung Medical Center, Sungkyunkwan University School of Medicine, Seoul, Korea

**Keywords:** recurrent B-cell lymphomas, clonality, immunoglobulin gene rearrangements, BIOMED-2 multiplex polymerase chain reaction

## Abstract

Immunoglobulin (Ig) gene rearrangements remain largely unmodified during the clonal expansion of neoplastic cells. We investigated the clonal relationships between lymphoma components at diagnosis and at relapse by analyzing Ig gene rearrangements. A BIOMED-2 multiplex polymerase chain reaction (PCR) assay was performed in 27 patients using formalin-fixed paraffin embedded tissues, with subsequent cloning and sequencing of the amplified Ig genes in 17 patients. All 27 cases of primary and corresponding relapsed tumors showed monoclonal rearrangements of the Ig genes by BIOMED-2 PCR. Whereas IgVH or IgVK fragment lengths were identical in 8/27 pairs (30%), fragment lengths differed in 19/27 pairs (70%). In 17 cases analyzed by sequencing, an identical VDJ gene rearrangement was confirmed in 4/4 pairs (100%) with the same fragment lengths and in 10/13 pairs (77%) with different fragment lengths. Four of 17 primary lymphomas had multiple VDJ rearrangements, and three of them showed an unrelated relapse. Unrelated relapse was observed in 1/8 mantle cell lymphomas, 1/5 diffuse large B-cell lymphomas, and a large B cell lymphoma developed in a patient with a small lymphocytic lymphoma. Unrelated relapses developed after a longer disease-free interval and tended to show poorer outcome compared with related relapse. In summary, relapse of a lymphoma from an unrelated clone is uncommon, but can occur in B-cell lymphomas. Clonal relationships should be determined by sequencing of the Ig genes, and not just by comparing the PCR product size.

## INTRODUCTION

Despite recent advances in therapeutic strategies for non-Hodgkin's lymphoma (NHL), a significant proportion of patients relapse, or their tumors are refractory to treatments [1]. One of the most important questions regarding relapses is whether they represent true, clonally related diseases or unrelated second lymphomas arising *de novo*. Because the management of both tumor types should be different, distinguishing between recurrent B-cell lymphomas and second primary lymphomas has clinical importance. In high-grade tumors, high-dose chemotherapy followed by autologous hematopoietic stem-cell transplantation has become the standard of care for patients with their first relapse of NHL [2]. On the other hand, a second de novo lymphoma might be treated with first-line therapy. However, it is not possible to distinguish such unrelated second lymphomas from clonally related lymphomas from clinical data and histopathology.

The immunoglobulin (Ig) gene sequence of B-lymphocytes consists of a variable and a constant region. The variable region in the heavy chain of Ig (IgH) is assembled from a large pool of variable (V_H_), diverse (D_H_), and joining (J_H_) segments by recombination during early B-cell differentiation in the bone marrow [3]. In the most immature B-cell precursors, the IgH gene remains in the germ line or is only joined by D_H_J_H_ segments [4, 5]. In late pro-B cells, V_H_ sequences join to the joined D_H_J_H_ sequences to complete the IgH rearrangement [4, 6]. Further recombination at the heavy chain (H) locus is prevented by a functional V_H_D_H_J_H_ (hitherto VDJ) rearrangement that also triggers rearrangements at the light chain (L) loci, most often a k type followed by a l type [4, 7]. Diversity is generated at the joints between each gene segment by the addition or deletion of nucleotides during recombination [8]. Through this process, each B-cell harbors unique VDJ genes, which are largely unmodified during clonal expansion and are used as natural clonality markers in B-lymphoproliferative disease. In the germinal center (GC), somatic hypermutation (SHM) occurs in already rearranged VDJ gene sequences of mature B cells to generate a diversified repertoire of antibodies [9, 10]. Several previous studies applied gene rearrangement analysis to investigate the clonal origin of metachronous lymphomas that develop in the same patient and reported that primary lymphomas and relapsed ones might or might not share a common clonal origin, irrespective of histopathology type and period of remission [11–14]. However, most of these studies were case reports, or used the fragment lengths of amplified VDJ gene as markers of clonality, but not exact V, D, or J gene sequences. Herein, to investigate the clonal identity of lymphoma cells in primary and corresponding relapsed tumors, we focused on VDJ rearrangements using BIOMED-2 multiplex polymerase chain reaction (PCR) assays in 27 pairs of matched primary and relapsed B-cell lymphomas together with sequencing of the amplified Ig genes.

## RESULTS

### Patients

The study cohort consisted of 27 patients who had both primary and corresponding recurrent tumor tissues available. Their median age at the diagnosis of a B-cell lymphoma was 63 years (range, 19–74 years). Histologically, 11 cases presented initially as a diffuse large B-cell lymphoma (DLBCL), eight as a mantle cell lymphoma (MCL), two as a marginal zone lymphoma, four as a follicular lymphoma (FL), and two as a small lymphocytic lymphoma (SLL). A change of phenotype in the relapsed tumor was observed in three patients. These were changes from SLL to DLBCL in one patient (case #3), and from FL, Grade 3a to FL, Grade 3a and DLBCL in two patients (cases #8 and #22).

Clinically, one patient had stage I disease, seven had stage II, six had stage III, and 11 had stage IV at presentation. Fifteen patients had one recurrence, nine had two recurrences, and three experienced three recurrences. Patients who relapsed at 3–12 months after receiving treatment for a primary B-cell lymphoma were considered as having an early relapse. There were early relapses in five patients (cases #1, #8, #16, #22, and #23), and late relapses in 22. The range of relapse-free interval was 4–12 months (median, 7.5 months) and 17–100 months (median, 33.5 months) in early and late relapse cases, respectively. The first-line treatment for the patients with primary B-cell lymphomas was according to standard protocols, and included R-CHOP-based regimens in most cases. Clinical details of the patients including the types of therapy administered are presented in Table 1.

**Table 1:** Clinical details in 27 cases of relapsed B-cell lymphoma analyzed by BIOMED-2 PCR

Case No.	Sex	Age at diagnosis	Site (primary/relapse)	Diagnosis (primary/relapse)	Stage	Therapy of primary lymphoma	Therapy of first relapse	Time to relapse (month)	Type of relapse	F/U duration after relapse	Outcome
1	M	61	Stomach/LN	DLBCL/DLBCL	IV	R-CHOP	ICE/DEXA	6	early	12	Dead
2	M	73	Stomach/LN	DLBCL/DLBCL	III	R-CHOP	ESHAOX	19	late	1	Dead
3*	M	63	LN/LN	SLL/DLBCL	IV	chlorambucil	R-CHOP, ESHAOX, ICE/DEXA	100	late	24	Dead
4*	F	48	LN/LN	DLBCL/DLBCL	III	CHOP	R-DHAP, Auto-PBSCT	20	late	132	Alive
5	M	19	Brain/Brain	DLBCL/DLBCL	IE	HD MTX/Procarbazine/VCR	ICE/DEXA	53	late	96	Alive
6*	M	72	LN/LN	MCL/MCL	III	CHOP	FND, Rituximab	65	late	61	Alive
7*	M	54	LN/LN	FL, grade1/FL, grade1	III	NA	NA	39	late	62	Alive
8*	M	68	LN/LN	FL, grade 3a/FL, grade 3a +DLBCL	IV	CHOP	NA	4	early	18	Dead
9	F	21	LN/LN	MZBCL/MZBCL	II	CVP	IMVP16-Pd	62	late	62	Alive
10*	M	54	Tonsil/BM	MCL/MCL	IIA	CHOP	Etoposide/MethylPd/HD Ara-C/oxaliplatin	38	late	11	Dead
11*	M	46	Ileocecum/LN	MCL/MCL	IV	CHOP	R-DHAP, Auto-PBSCT	37	late	16	Dead
12	M	48	LN/LN	FL, grade1/FL, grade1	III	Chlorambucil	Chrolambucil	63	late	49	Alive
13*	F	71	Cecum/Bone	DLBCL/DLBCL	IIA	R-CHOP	DHAP	17	late	9	Dead
14*	M	74	LN/Stomach	MCL/MCL	IV	Fludarabine,R-CVP	Etoposide/MethylPD/HD Ara-C/oxalipatin	31	late	4	Dead
15*	M	62	Tonsil/LN	MCL/MCL	IIA	R-CHOP	DHAP	28	late	68	Dead
16*	M	74	Ileum/LN	MCL/MCL	IV	Rituximab/CHOP	DHAP, Auto-PBSCT	12	early	37	Alive
17*	M	60	Cecum/BM	DLBCL/DLBCL	IV	R-CHOP	DHAP, Auto-PBSCT	68	late	1	Dead
18	F	66	Tonsil/LN	DLBCL/DLBCL	II	R-CHOP	ICE/DEXA	43	late	30	Alive
19	M	57	LN/Tonsil	DLBCL/DLBCL	II	R_CHOP	GIDOX salvage chemotherapy, ESHAOX	40	late	10	Alive
20*	M	71	Forearm/LN	MCL/MCL	III	R-Hyper CVAD	FND, Ibrutinib	49	late	33	Alive
21*	M	54	Tonsil/Tonsil	MCL/MCL	IV	R-CHOP	DHAP, Auto-PBSCT	28	late	39	Alive
22*	M	47	LN/Tonsil	FL, grade 3a/FL, grade 3a +DLBCL	II	CHOP	FND, Rituximab	9	early	18	Alive
23	M	39	LN/LN	DLBCL/DLBCL	IVA	R-CHOP	Rituximab+DHAP, Auto-PBSCT	9	early	56	Alive
24*	M	53	LN/LN	DLBCL/DLBCL	IVA	RCHOP	ESHAOX	21	late	27	Alive
25*	M	69	LN/LN	DLBCL/DLBCL	IVA	RCHOP	ICE/DEXA	20	late	8	Dead
26	M	69	LN/Parotid gland	MZBCL/MZBCL	NA	CHOP	NA	33	late	120	Dead
27	M	65	LN/LN	SLL/SLL	NA	CVP	NA	48	late	0	Dead+

* Cases further analyzed by sequencing ^+^Dead due to another disease

Abbreviations: M, male; F, female; LN, lymph node; DLBCL, diffuse large B-cell lymphoma; SLL, small lymphocytic lymphoma; MCL, mantle cell lymphoma; FL, follicular lymphoma; MZBCL, marginal zone B-cell lymphoma; NA, not available.

### BIOMED-2 multiplex PCR assays

Monoclonal gene rearrangements were identified if one or two prominent peaks on a background of polyclonal peaks were present in at least one PCR tube. The primary and relapsed B-cell lymphomas had clonality for IgH in 26 (96%) and 25 (93%) cases, respectively, and for IgK in 25 (93%) and 24 (89%) out of the 27 cases, respectively. When combining the complete set of BIOMED-2 reactions (IGH tubes A, B, and C; IGK tubes A and B), all 27 cases of primary and corresponding relapsed tumor cells showed monoclonal rearrangements of the Ig genes. We then compared the lengths of Ig gene rearrangement products in the 27 paired samples. When the Ig V–H or Ig V–K fragment lengths in at least one tube (IGH tubes A, B, and C; IGK tubes A and B) were the same size, they were considered as having identical fragment lengths. Products of IgH PCR amplifications for tubes A, B, and C were compared between the primary and corresponding relapsed tumor cells in eight, two, and 14 pairs, respectively. In three pairs, the Ig V–K gene junction was compared between primary and corresponding relapsed tumor cells. In eight cases (30%), the Ig V–H or Ig V–K fragment lengths in at least one tube were identical in size, whereas fragment lengths were different in 19 cases (70%). Of these 19 cases showing different fragment lengths, 15 showed a difference of less than 5 bp and four showed a difference of more than 5 bp.

### Sequencing of Ig genes

Examination of the Ig rearrangements in primary and corresponding relapsed tumors yielded the same-sized PCR products in eight cases. To investigate this further, we performed cloning and sequencing of the PCR products in 19 cases that showed strong bands in gel electrophoresis. The BIOMED-2 PCR products covering the junctions at IgVH FR1–VDJ, IgVH FR2–VDJ, IgVH FR3–VDJ, and Ig VK–VDJ were analyzed in one, two, 13, and one pair, respectively. In 17/19 patients, the IgH or IgK gene could be sequenced successfully for both primary and corresponding relapsed tumors. Pairs for two of the patients (cases #26 and #27) could not be sequenced because the DNA was of insufficient quantity or quality for analysis.

### Clonal relationships

**Figure 1:**
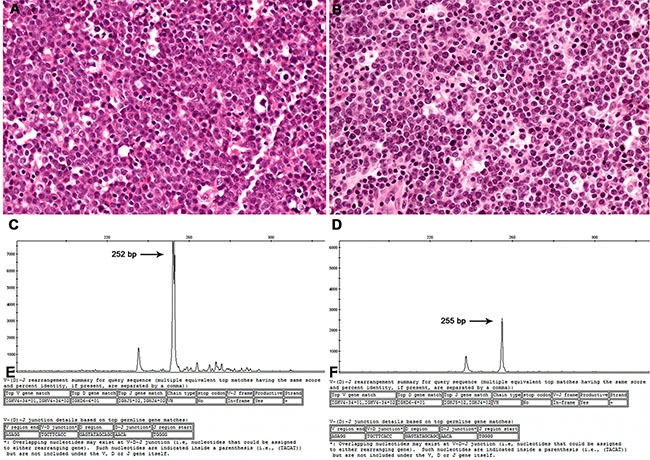
Representative case (#4) Histopathology sections of a lymph node diagnosed as a DLBCL in both 2002 (**A**) and 2004 (**B**). Using GeneMapper analysis, a peak at 252 bp was seen in the primary DLBCL (**C**), whereas a peak at 255 bp appeared in the relapsed DLBCL (**D**). In sequencing analysis, both tumors showed identical V_H_, D_H_, and J_H_ gene segments and junctions (**E** and **F**).

**Figure 2:**
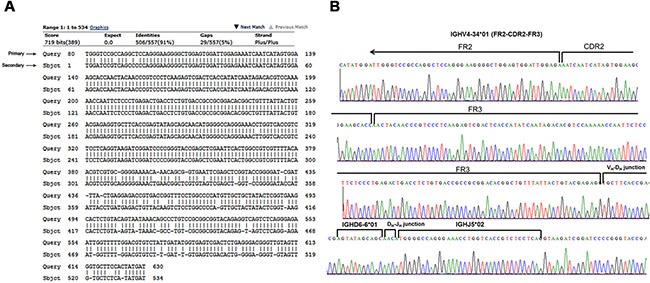
Nucleotide sequence of the rearranged Ig V_H_4–34 genes cloned from a primary tumor and relapse (case #4) (**A**) Both primary and relapsed B-cell lymphoma cells showed 91% sequence identity after shared VDJ rearrangements, and alignment of the completely sequenced region. (**B**) The IGH segment usages identified are indicated above the nucleotide residues.

**Figure 3:**
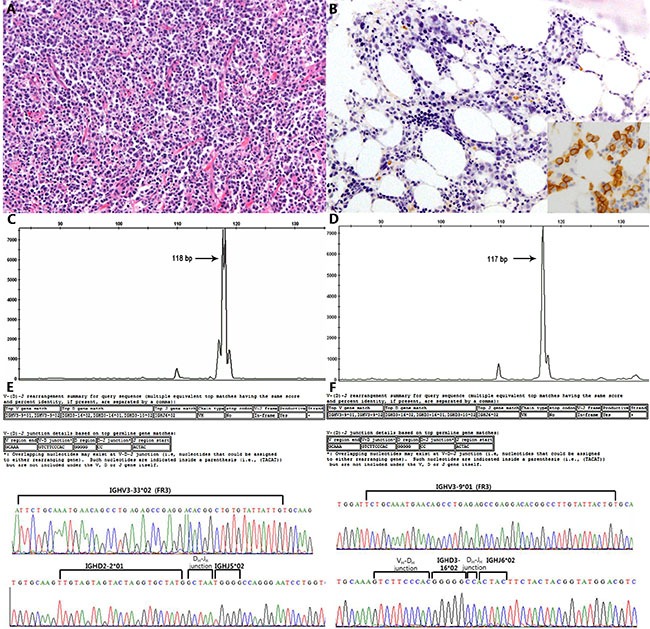
Representative case (#17) of an unrelated relapse from a DLBCL to a DLBCL (**A** and **B**) Different IGH fragment lengths (difference of one base pair) were found in the GeneMapper analysis (**C** and **D**), and different V_H_, D_H_, and J_H_ gene segments and junctions were found by sequencing (**E** and **F**).

Table 2 shows the V, D, and J gene segment usage in the primary and corresponding relapsed tumors. Primary and corresponding relapsed B-cell lymphomas showed identical V_H_, D_H_, and J_H_ gene segments in 10/13 cases showing different fragment sizes, as well as in four cases with the same fragment sizes. Taken together, 14/17 (82%) primary and corresponding relapsed tumors were clonally related, whereas three (18%) pairs were clonally unrelated. Most of the clonally related tumors showed identical fragment lengths (4/14) or just one nucleotide difference (7/14). Otherwise, two pairs (cases #4 and #15) and one pair (case #22) showed three and two nucleotide differences, but both tumors showed identical V_H_, D_H_, and J_H_ gene segments and junctions (Figures 1 and 2). Three pairs of clonally unrelated tumors showed one nucleotide differences in two and two nucleotide difference in one. All three pairs had different V_H_, D_H_, and J_H_ gene segments, and junctions (Figure 3).

**Table 2:** Sequencing analyses of the BIOMED-2 PCR product in 17 cases of relapsed B-cell lymphoma

Case No.	Diagnosis (primary/relapse)	Amplicon size (primary/relapse)	Top V gene match (primary/relapse)	Top D gene match (primary/relapse)	Top J gene match (primary/relapse)	FR3/CDR3 gene Homology (%)	Clonal relationship	Number of distinct VDJ rearrangements/total colonies sequenced
3	SLL	131	IGHV7-4-1*03	IGHD2-2*02,IGHD2-2*01,IGHD2-2*03	NA	100.0	Unrelated	2/4
	DLBCL	130	IGHV1-69*06,IGHV1-69*13,IGHV1-69*09	IGHD6-13*01	IGHJ5*02	96.0		2/4
20	MCL	141	IGHV3-48*03, IGHV3-69-1*02	IGHD6-19*01, IGHD6-25*01, IGHD1-26*01	IGHJ6*02	100.0	Related	
	MCL	140	IGHV3-48*03, IGHV3-69-1*02	IGHD6-19*01, IGHD6-25*01, IGHD1-26*01	IGHJ6*02	100.0		
21	MCL	130	IGHV3-23*01, IGHV3-23D*01, IGHV3-23*02	IGHD1-1*01	IGHJ4*02	100.0	Related	
	MCL	130	IGHV3-23*01, IGHV3-23D*01, IGHV3-23*02	IGHD1-1*01	IGHJ4*02	100.0		
14	MCL	107	IGHV4-34*05,IGHV4-34*01,IGHV4-34*02	IGHD4-23*01	IGHJ4*02	100.0	Related	1/3
	MCL	107	IGHV4-34*05,IGHV4-34*01,IGHV4-34*02	IGHD4-23*01	IGHJ4*02	100.0		1/1
15	MCL	257	IGHV3-53*01, IGHV3-53*02	IGHD3-10*01	IGHJ4*02	100.0	Related	1/4
	MCL	260	IGHV3-53*01, IGHV3-53*02	IGHD3-10*01	IGHJ4*02	100.0		1/1
16	MCL	137	IGHV3-9*01, IGHV3-9*02	IGHD4-17*01	IGHJ6*02	100.0	Related	
	MCL	137	IGHV3-9*01, IGHV3-9*02	IGHD4-17*01	IGHJ6*02	100.0		
10	MCL	111	IGHV4-34*05, IGHV4-34*01, IGHV4-34*12	IGHD4-11*01, IGHD4-4*01	IGHJ4*02	100.0	Unrelated	4/4
	MCL	109	IGHV4-34*05, IGHV4-34*05, IGHV4-34*01	IGHD6-6*01	IGHJ5*02, IGHJ4*02	87.5		2/4
11	MCL	121	IGHV3-33*02	IGHD4-23*01	IGHJ4*02	100.0	Related	
	MCL	120	IGHV3-33*02	IGHD4-23*01	IGHJ4*02	100.0		
6	MCL	132	IGHV3-33*02	IGHD6-6*01	IGHJ6*02	100.0	Related	1/2
	MCL	133	IGHV3-33*02	IGHD6-6*01	IGHJ6*02	100.0		3/4
7	FL, grade1/	236,282	IGKV3D-7*01,IGKV3-7*04,IGKV3-7*02		N/A	95.7	Related	3/4
	FL, grade1/	236,282	IGKV3D-7*01,IGKV3-7*04,IGKV3-7*02		N/A	100.0		2/5
8	FL, grade 3a	132	IGHV4-4*07, IGHV4-39*06, IGHV4-4*02	IGHD3-16*02, IGHD3-16*01, IGHD6-13*01	IGHJ6*02	100.0	Related	
	FL, grade 3a +DLBCL	133	IGHV4-4*07, IGHV4-39*06, IGHV4-4*02	IGHD3-16*02, IGHD3-16*01, IGHD6-13*01	IGHJ6*02	100.0		
22	FL, grade 3a	131	IGHV3-23*01, IGHV3-23D*01, IGHV3-23*02	IGHD1-1*01	IGHJ4*02	100.0	Related	
	FL, grade 3a +DLBCL	133	IGHV3-23*01, IGHV3-23D*01, IGHV3-23*02	IGHD1-1*01	IGHJ4*02	100.0		
4	DLBCL	252	IGHV4-34*01, IGHV4-34*02	IGHD6-6*01	IGHJ5*02, IGHJ4*02	87.5	Related	1/4
	DLBCL	255	IGHV4-34*01, IGHV4-34*02	IGHD6-6*01	IGHJ5*02, IGHJ4*02	87.5		1/4
13	DLBCL	145	IGHV1-NL1*01	IGHD2-8*02	IGHJ6*02	100.0	Related	
	DLBCL	146	IGHV1-NL1*01	IGHD2-8*02	IGHJ6*02	100.0		
17	DLBCL	118	IGHV3-33*02	IGHD2-2*01, IGHD2-2*03	IGHJ5*02, IGHJ4*02	80.0	Unrelated	3/4
	DLBCL	117	IGHV3-9*01, IGHV3-9*02	IGHD3-16*02, IGHD3-16*01, IGHD3-10*02	IGHJ6*02	100.0		2/3
24	DLBCL	146	IGHV3-11*05,IGHV3-23*01,IGHV3-7*03	IGHD3-3*02,IGHD3-3*01	IGHJ4*02	100.0	Related	
	DLBCL	145	IGHV3-11*05,IGHV3-23*01,IGHV3-7*03	IGHD3-3*02,IGHD3-3*01	IGHJ4*02	100.0		
25	DLBCL	312	IGHV3-48*02	IGHD5-24*01	IGHJ4*02	100.0	Related	
	DLBCL	311	IGHV3-48*02	IGHD5-24*01	IGHJ4*02	100.0		

Abbreviations: DLBCL, diffuse large B-cell lymphoma; SLL, small lymphocytic lymphoma; MCL, mantle cell lymphoma; FL, follicular lymphoma.

### Somatic hypermutation

Compared with the closest germ line sequence of FR3 and CDR3 in the Ig blast database, 3/17 primary cases (18%) and 3/17 corresponding relapsed cases (18%) showed mutated Ig V genes (IGV). Mutated IGV was observed in 1/3 primary FLs, 2/5 primary DLBCLs, whereas all 8/8 primary MCL cases showed an unmutated IGV. Of these, 2/14 clonally related primary cases (14%) and 1/3 unrelated cases (33%) showed a mutated IGV. Among unrelated relapses, one patient with an unmutated SLL relapsed with a mutated DLBCL and the other patient with an unmutated MCL relapsed with a mutated MCL. One patient with a mutated DLBCL relapsed with an unrelated DLBCL with a mutated IGV gene. The results are summarized in Table 3.

**Table 3:** Clonal relationship correlated with IGV gene mutation and VDJ heterogeneity of primary lymphoma

Primary	Relapse			
		Clonally Related (*n* = 14)		Clonally unrelated (*n* = 3)
IgV gene unmutated (*n* = 14)		(*n* = 12)		(*n* = 2)
	MCL/MCL	7	SLL/DLBCL	1
	FL/FL	2	MCL/MCL	1
	DLBCL/DLBCL	3		
IgV gene mutated (*n* = 3)		(*n* = 2)		(*n* = 1)
	DLBCL/DLBCL	1	DLBCL/DLBCL	1
	FL/FL	1		
Heterogeneous VDJ rearrangement (*n* = 4)		(*n* = 1)		(*n* = 3)
	FL	1	SLL/DLBCL	1
			MCL/MCL	1
			DLBCL/DLBCL	1
Homogeneous VDJ rearrangement (*n* = 4)		(*n* = 4)		
	MCL	3		
	DLBCL	1		

Abbreviations: DLBCL, diffuse large B-cell lymphoma; SLL, small lymphocytic lymphoma; MCL, mantle cell lymphoma; FL, follicular lymphoma.

### Heterogeneous VDJ rearrangements

Of 17 pairs confirmed by sequencing, more than two colonies were analyzed in eight primary tumors. Among them, three tumors harbored from two to four distinct VDJ rearrangements (cases #3, #7, #10, and #17; Table 2). If the primary and corresponding relapsed tumors harbored at least one of the same VDJ rearrangements, they were considered to be clonally related. Three of four cases of primary tumors with multiple VDJ rearrangements relapsed with an unrelated second lymphoma (cases #3, #7, and #17).

### Clinicopathological parameters correlated with clonal relationship

In 17/27 patients, we confirmed the clonal relationship of both primary and corresponding relapsed tumors by sequencing. Unrelated relapse accounted for 1/8 MCLs, 1/5 DLBCLs, and one DLBCL developed in a patient with SLL. All three FLs, 7/8 MCLs, and 4/5 DLBCLs showed related relapses. Because there were only three unrelated relapses, comparisons of clinical characteristics between cases of unrelated and related relapses were difficult. However, the time to relapse tended to be longer in cases of unrelated relapses compared with related relapses. There was no significant association between clinical stage and clonal relationship. One patient with an unrelated DLBCL relapse died 1 month after relapse whereas the median survival of patients with related DLBCL relapses was 18 months. Likewise, the patient with an unrelated MCL relapse showed poor survival compared with patients with related relapse (Supplementary Table 1).

## DISCUSSION

This study aimed to examine evidence of clonal relationships between primary and corresponding relapsed tumors, based on analysis of the rearranged Ig sequences. The VDJ rearrangement is unique in length and sequence for each B-cell type and is highly conserved during the clonal expansion of neoplastic B-cells [15, 16]. Therefore, the identification of clonality based on Ig gene rearrangement has been utilized in several studies after the discovery of these rearrangements [12, 14, 17–22]. The traditional PCR design with one pair of primers to detect an Ig rearrangement yields frequent false-negative results because the primers cannot cover extensive sequence variations. In particular, malignant tumors derived from the GC, or of post-GC origin including DLBCLs harboring somatically hypermutated VH regions, show lower rates of detection of clonality when using PCR-based assays [23]. In 2003, a large European collaborative group developed the BIOMED-2 assays using a multiplex PCR approach including multiple pairs of consensus primers [23]. This BIOMED-2 assay can detect clonality in 99% of NHL tumors [24]. The most useful tests for suspected B-cell malignancies are assays that detect complete IGH rearrangements and IGK rearrangements [24]. In our results, the combination of IGH and IGK BIOMED-2 assays revealed high sensitivity for identifying monoclonality.

Many practicing clinicians and pathologists assume that when clonality studies performed on metachronous tumors yield different fragment lengths they must be from different tumors. This study clearly shows that this is not the case. A GeneMapper analysis showed that 10/13 pairs (77%) showing different fragment sizes between the primary and corresponding relapsed tumors were clonally related. Such a difference in fragment size in clonally related tumors is caused by somatic mutations in already rearranged V gene sequences in the GC [9, 10]. Somatic mutation can occur in any region of IgV genes from the FR1 to CDR3 regions. Although the VDJ segments are same in both the primary and corresponding relapsed B-cell lymphomas, there can be differences in base pair numbers between pairs for the abovementioned reasons. Therefore, it is insufficient to determine clonal relationships by comparing the Ig fragment size alone between primary and corresponding relapsed B-cell lymphomas.

By sequencing the IgV gene, we confirmed a clonal relationship in 17 pairs of 27 primary and corresponding relapsed tumors, of which 14 pairs (82%) were clonally related. Whereas three pairs (18%) were clonally unrelated, two of them showed the same histopathology type of B-cell lymphoma. In other words, a second lymphoma with the same histopathology characteristics as the first one is not always indicative of a relapse of the original clone.

Previous studies sequencing the IgV genes demonstrated that recurrent B cell lymphomas with different types of histopathology were derived from distinct B-cell clones. These included follicular lymphomas with an MCL relapse [18], SLLs with a relapse with DLBCL [25], DLBCLs with a FL relapse [12], and FLs with a BL relapse [26]. In our study, a DLBCL that developed in a patient with an SLL also showed a distinct VDJ rearrangement. However, a relapse with a different histopathology is not always derived from a distinct B-cell clone. There have been reports documenting the same clonal origin for a lymphoplasmacytic lymphoma with a DLBCL relapse [19] and an SLL with a DLBCL relapse [27].

Several studies on the genetic evolution of B-cell tumors have provided clues for understanding the molecular mechanisms of relapse using high-throughput sequencing [15, 28]. Jiang *et al.* [15] demonstrated the evolutionary patterns for 14 DLBCL relapses by high-throughput sequencing of rearranged Ig genes and exome sequencing. They found all except one case harbored the same VDJ rearrangements, demonstrating that they were clonally related. In these clonally related diagnosis and relapse tumors, unique subclones with distinctive somatic hypermutation patterns within the dominant VDJ sequence were found. By phylogenetic analysis of these SHM profiles of major VDJ rearrangements, they suggested that early divergent tumor is derived from a very minor subclone arising earlier in the diagnosis tumor and associated with higher clonal heterogeneity, while late divergent tumor is derived from a subclone that is more closely related to the major clone in diagnosis tumor. Unrelated relapse of diffuse large B cell lymphoma with a distinct VDJ rearrangement from diagnosis tumor is not common and has been reported in only 1 of 14 cases by Jiang et al. [15], 2 of 13 cases by de Jong et al. [20] and 2 of 14 cases by Geurts-Giele et al. [21]. Taken together, overall 14.5% of DLBCLs including 3 of 14 cases in our study relapse from a distinct clone. While related relapse arises through somatic hypermutation in preexisting clone, cellular origin of unrelated relapse in diffuse large B cell lymphoma is not well investigated. In Hodgkin lymphoma in Richter transformation, HRS or HRS-like cells in unrelated relapse is commonly positive for Epstein-Barr virus, suggesting an underlying immunodeficiency present in B-CLL patients may increase the risk of an EBV infection that in turn fosters an environment in which secondary neoplasms are more likely to occur.[22, 29] Accordingly a variety of regulatory abnormalities of T cells and autoimmune-associated phenomena are frequently observed in patients with CLL [30].

In our study, we found that four primary lymphoma samples harbored two to four distinct VDJ rearrangements. Multiple VDJ rearrangements in a single tumor may represent biallelic rearrangement or multiple clones or contaminated normal cells [31, 32]. Of note is that all the three unrelated relapsed lymphomas had heterogeneous VDJ rearrangements in the primary tumors. From this finding, one can assume that the clonally unrelated relapses arose from minor clones, which were not detected at the initial presentation by gene rearrangement analysis using simple sequencing technology. However, this speculation should be validated by further experiment. Targeted deep-sequencing technology would make up for the defects in current techniques in detecting minor clones.

It has been reported that the mutational status of the IGV gene may be a prognostic marker. In patients with a chronic lymphocytic leukemia, it has been shown that those with somatically mutated IGV sequences have a better prognosis than among unmutated cases. In MCL, known to arise from pre-GC cells of the mantle zone, studies have demonstrated that somatic hypermutation can be detectable in up to one-third of the cases when using < 98% homology as the cutoff [33, 34]. Although, the clinical relevance of the mutational status of the IGV gene has been controversial, molecular subsets of MCLs defined by IGHV mutational status have distinct clinical features [34]. In our study, we analyzed the mutational status of the FR3 and CDR3 regions of IgV genes. The frequency of somatic hypermutation in FL and DLBCL was a little lower than that reported previously [35], that may be because we analyzed FR3 and CDR3 region only. Two of five cases with a primary DLBCL carried a mutated IGV sequence, whereas all cases with a primary MCL showed an unmutated IGV. There were no significant differences in the IGV mutational status according to clonal relationships among the cases studied here.

Our findings that not all relapsed B-cell lymphomas are true recurrences arising from the major clone of the original lymphoma might have clinical significance for treatment strategies. While salvage therapy followed by bone marrow transplantation improves the outcome for patients with a relapsed lymphoma, some of them are not eligible for further therapy because of age or refractoriness to chemotherapy. In our study, two patients, one with an unrelated DLBCL and one with an MCL, died of disease shortly after diagnosis. Because the current therapy for recurrent lymphoma does not consider the possibility of dealing with a second, clonally unrelated lymphoma, it would be interesting to see how these unrelated lymphomas respond to chemotherapy and correlate the clonal relationship with clinical outcome in a larger number of cases. The results might merit consideration in future clinical studies to determine whether a less-aggressive treatment might be justifiable in patients with relapsed unrelated lymphomas.

In conclusion, we confirmed that a few relapsed B-cell lymphomas arise from an unrelated clone by sequencing the Ig genes, not just by comparing the PCR product size. The therapeutic strategies for patients with these groups of clonally unrelated lymphomas need to be established in prospective studies with enrollment of a larger number of cases.

## MATERIALS AND METHODS

### Samples

Fifty-four formalin-fixed paraffin wax-embedded (FFPE) sets of tissue from 27 patients who had both primary and corresponding relapsed tumors were studied. All cases were confirmed pathologically, and diagnosed based on histopathology and immunohistochemical criteria as detailed in the 2008 World Health Organization classification of lymphoid neoplasms. Hematoxylin and eosin-stained slides were reviewed and representative tumor blocks were used. This study was performed according to a protocol approved by the Samsung Medical Center Institutional Review Board in accordance with the Declaration of Helsinki (2014–08–106).

### Multiplex PCR analysis for Ig gene rearrangements

For FFPE specimens, two 5-μm sections were deparaffinized with xylene and washed three times with 100% ethanol. Genomic DNA was extracted using ReliaPrep™ FFPE gDNA extraction kits (Promega, Madison, WI, USA) following the manufacturer's instructions. Genomic DNA quality was assessed using BIOMED-2 control gene PCR assay kits (*In Vivo* Scribe Technologies, San Diego, CA, USA), NanoDrop 1000 (Thermo Scientific, Wilmington, DE, USA) and Quantus (Promega). Samples with a DNA product size of ≥ 200 base pairs (bp) were analyzed using BIOMED Ig primers. Those with a DNA product size of ≤ 100 bp or considered inadequate were excluded. B-cell clonality was determined in duplicate with the full set of BIOMED-2 assays, which include five reactions targeting IGH (IGHA, FR1 [variable region framework 1]–J; IGHB, FR2–J; IGHC, FR3–J; IGHD, D1–6–J; IGHE, D7–J), two reactions targeting IGK (IGKA, V–J; IGKB, V–Kde and JC intron–Kde), and one reaction for IGL (V–J). PCR assays were run in duplicates, and polyclonal and monoclonal controls were included for each experiment. PCR products were heteroduplex treated (denatured at 95°C for 10 min) and rapidly transferred to 4°C for 60 min to promote duplex formation. Positive (monoclonal) and negative (polyclonal) controls in addition to a blank control (H_2_O) were included in all cases. Gene scanning was carried out on an ABI Prism 3130Xl Sequencer (Applied Biosystems, Foster City, CA, USA) using GeneMapper Analysis Software, version 4.0. PCR products that fell within the valid size range and were at least three times the amplitude of the third largest peak in the polyclonal background were considered as positive peaks. The results were scored “monoclonal” if the IGH V–J, IGK V–J, and IGL V–J multiplex PCRs yielded a reproducible clonal product at the same position in both replicates. If the apparent clonal peaks were of different fragment lengths in the two replicates (pseudoclonality), the analysis was scored “not evaluable.”

### Sequencing analysis of the clonal rearranged immunoglobulin genes

Clonal PCR products of the IGH V–J and IGK V–J reactions were sequenced to define IGHV (or *IGKV*), IGHD, and *IGHJ* (or *IGKJ*) gene and allele usage, the percentage of identity to the closest germline *IGHV* allele, complementarity-determining region (CDR)-3 length and composition. For sequencing, dominant and strong bands from 2% agarose gels were purified using LABOPASS Gel extraction kits (Cosmogenetech, Seoul, Republic of Korea), ligated into the pMD20-T vector (Takara Bio Inc., Ōtsu, Japan) and transformed into Top10 competent cells (Invitrogen, Carlsbad, CA, USA). The transformed cells were selected on LB ampicillin agar plates containing X-gal and isopropyl b-D-1-thiogalactopyranoside (IPTG). White colonies were screened using PCR with vector primers (M13 forward/reverse). PCR products showing the expected insert sizes were sequenced in both directions using an ABI sequencer with BigDye Terminator v3.1 Cycle Sequencing kits (Applied Biosystems, Foster City, USA). Except for one sample, at least two colonies from each sample were sequenced.

Ig gene usage and somatic hypermutations were analyzed using the Ig BLAST database (http://www.ncbi.nlm.nih.gov/igblast/). Hypermutation frequencies in FR3 and CDR3 were calculated based on the absolute number of nucleotides in FR3 and CDR3 regions as defined by IgBLAST. Based on the rate of somatic mutations detected in these genes, cases were classified as “unmutated” or “mutated.” Compared with the most similar germline sequence, “unmutated” genes were defined as those with < 2% differences, while “mutated” genes were those with ≥ 2% difference from the closest germline sequence.

## SUPPLEMENTARY MATERIALS TABLE


