# Supplementary material for: Clonal relationships in recurrent B-cell lymphomas
Source: Oncotarget. 2016 Feb 2;7(11):12359–71. doi: 10.18632/oncotarget.7132 (PMC4914290; doi:10.18632/oncotarget.7132)
Supplement: Supplementary file 1 [file oncotarget-07-12359-s001.pdf]

Clonal relationships in recurrent B-cell lymphomas

Supplementary Materials

Supplementary Table S1: Clinical characteristics correlated with clonal relationship

Clinical characteristics		Related (n = 14)	Unrelated (n = 3)	P-value
Stage	II	3 (21.4%)	1 (33.3%)	1
	III	4 (28.6%)	0 (0%)	
	IV	7 (50.0%)	2 (66.7%)	
Time to relapse (month)	Median (range)	24.5 (4–71)	68 (38–100)	0.032*
Type of relapse	early	3 (21.4%)	0 (0%)	1
	late	11(78.6%)	3 (100.0%)	
Median survival after relapse (month)				
	FL (n = 3)	40	NC	
	CLL/ DLBCL (n = 1)		24	
	MCL/ MCL (n = 8)	37	11	0.5*
	DLBCL/ DLBCL (n = 5)	18	1	0.4*
Clinical outcome	Alive	8 (57.1%)	0 (0%)	0.576
	Dead	6 (42.9%)	3 (100%)	

*Mann-Whitney test
Abbreviations: DLBCL, diffuse large B-cell lymphoma; SLL, small lymphocytic lymphoma; MCL, mantle cell lymphoma; FL, follicular lymphoma; NC, non-checkable.
